# Identifying the sociodemographic determinants of subjective health complaints in a cross-sectional study of Greek adolescents

**DOI:** 10.1186/1744-859X-11-17

**Published:** 2012-06-29

**Authors:** Dimitra Petanidou, George Giannakopoulos, Chara Tzavara, Christine Dimitrakaki, Ulricke Ravens-Sieberer, Gerasimos Kolaitis, Yannis Tountas

**Affiliations:** 1 Centre for Health Services Research, Department of Hygiene, Epidemiology and Medical Statistics, Athens University Medical School, Athens, Greece; 2 Department of Child Psychiatry, Athens University Medical School, ‘Aghia Sophia’ Children's Hospital, Athens, Greece; 3 Robert Koch Institute, Child and Adolescent Health, Berlin, Germany

**Keywords:** Adolescence, Financial resources, Sociodemographic factors, Socioeconomic status, Subjective assessment, Subjective health complaints

## Abstract

**Background:**

Experience of common health symptoms without a clear physical or psychological cause, such as headache or dizziness, is often reported in adolescence. The present study attempted to investigate associations of self-reported subjective health complaints (SHC) with a number of sociodemographic factors of Greek adolescents.

**Methods:**

Questionnaires were administered to a Greek nationwide random school-based sample of adolescents aged 12 to 18 years and their parents in 2003. Data from 922 adolescent-parent pairs were analyzed (response rate = 63%). Adolescents’ reported subjective health complaints were assessed for their association with a number of sociodemographic factors: age, sex, type of area of residence according to level of urbanization, immigration background, parental education and employment status, family socioeconomic status and perceived quality of financial resources (PQFR). Multiple linear regression analysis was used to assess the association of the aforementioned factors with subjective health complaints as the dependent variable.

**Results:**

Most sociodemographic variables, apart from area of residence and immigration background, were independently associated with subjective health complaints in the univariate analyses. The multiple linear regression analysis, however, limited the factors that could predict adolescents’ subjective health complaints to four (age, sex, Family Affluence Scale score and perceived quality of financial resources). Some considerations regarding parental employment status and immigration background are highlighted.

**Conclusions:**

Our study highlights the sociodemographic components of subjective health complaints in the Greek adolescent population. The need to include adolescent-specific measures when collecting information on adolescents’ social background is underlined. Identifying vulnerable adolescent populations could lead to effective health promoting and preventive interventions.

## Background

Despite the fact that adolescence is widely considered as an age period of good health in Western societies, a respectable amount of scientific interest has been placed on research into adolescents’ subjective health complaints [1]. Subjective health complaints (SHC) is a general term used to describe a variety of common health symptoms, for example, headache, abdominal pain, fatigue, nervousness, dizziness and so on, experienced by the individual without pathological signs or where the pathological findings are disproportionate to the illness experience [2]. The terms ‘medically unexplained’, ‘functional’ and ‘psychosomatic’ have also been employed in the literature to describe the same constellation of symptoms. However, the term ‘subjective health complaints’ is preferable for its neutral qualities, since it allows no assumptions on the etiology of symptoms and it makes no supposition about the direction of the causal link between biological and psychological factors. What is more, it offers the opportunity to bring together and elaborate on possible contributions to the experience of symptoms, involving personality characteristics (for example, temperament), psychological mechanisms (such as coping and attachment type), cognitive aspects (for example, cognitive activation theory of stress), family influences (for instance, parental psychopathology) and psychosocial factors (in example, relationships with peers, quality of social context), while, at the same time, it permits examination of possible associations with other psychological symptoms and disorders, such as depression, anxiety and somatoform disorders [1,3].

SHC have been documented as a public health concern in the adult non-clinical population [4]. Moreover, high rates of SHC have been reported among children and adolescents from several European countries over the last decade [5], suggesting that they may constitute a significant public health issue across the human lifespan. Experience of recurrent health complaints without a clear physical or psychological cause has been associated with poor mental health [6] and decreased well-being and functional ability [7]. What is more, existing research demonstrates that an increased level of health complaints is related to lower academic performance and absenteeism [8], increased demand for primary care services and increased use of medicine [6,8-10].

Defining the sociodemographic factors that are related to self-reported complaints about somatic and psychological health in adolescence and understanding the underlying relationships is critical for planning both early preventive actions in community settings and effective multidisciplinary clinical interventions. Better understanding of SHC could be a useful tool for health professionals in their effort to fully comprehend and respond to adolescents with vague symptoms effectively.

The most well established and constant finding across relevant cross-national studies is that SHC are both gender and age dependent, indicating that females tend to complain more about their health and that complaints increase with age for both genders [7,8,11,12]. Nevertheless, not all sociodemographic factors have resulted in clear findings regarding their association with adolescents’ SHC. The type of area of residence according to level of urbanization ( that is, urban, semi-urban and rural) is one such example. Evidence from the field of mental health indicate that high levels of stress and mental health symptoms have been documented among both rural [13] and urban adolescents [14]. In the same vein, the hypothesis that adolescents with an immigration background are at greater risk of mental health and emotional/behavioral problems has not been consistently confirmed in research literature [15,16]. Therefore, drawing any general or sound conclusions over the effect of immigration on adolescents’ general and/or mental health still appears to remain a challenge.

Regarding the socioeconomic gradient of health assessment in adolescence, research so far has led to contradictory findings. There have been studies demonstrating evidence that adolescent health outcomes do not differ across socioeconomic status (SES) [17,18], while other studies support the impact of socioeconomic inequalities on adolescents’ health, even to a greater extent comparing to childhood [19,20]. This contradiction reflects the variation of health outcomes measured as well as the complexity and variety of indicators that are incorporated in the widely utilized variable of socioeconomic status [21].

Previous studies on adolescent population have commonly used parental indicators of socioeconomic status, namely parental education and parental employment status, in order to define the family socioeconomic background [19,22,23], as they reveal the families’ financial and cultural resources [24]. However, since adolescence is an era of gradual differentiation from parental influences, it has been argued that parent-based measures of socioeconomic position may not be accurate in catching health inequality during the whole spectrum of adolescence and should, therefore, be interpreted with caution [24,25].

Studies on adolescent population have shifted attention from parental indicators of socioeconomic background to adolescents’ assessments of socioeconomic status. In children’s and adolescents’ self-reports, the Family Affluence Scale (FAS) [26] is the measure of preference since it is age appropriate and reflects the material well-being of the individual’s family [24,27]. The employment of FAS in the investigation of adolescent health outcomes has revealed inequalities in a wide spectrum of different health measures [27], such as self-rated health, life satisfaction, health related quality of life [28] and SHC [5,29].

Current research has lately encompassed the individual’s subjective perception of socioeconomic status, as a factor that involves a wide range of both social and economic experience and appears to be a good predictor of health for both adults and adolescents [30-32]. Adolescents’ subjective assessments of socioeconomic status have introduced ‘subjective’ indicators in the form of self-reported scales of subjective perception of social status, familial financial difficulties and available financial resources. Research so far has argued that incorporating individuals’ subjective perception of socioeconomic status could serve as an additional useful tool in the effort to examine socioeconomic inequalities in adolescent health [23,30,32]. Adolescents’ perceived quality of their financial resources (PQFR) aims to explore whether the adolescent feels that he/she has enough financial resources to lead a lifestyle which is comparable to other adolescents and provides the opportunity to do things together with peers [33]. Since it has been only recently that studies have been shedding light on this direction, we decided to extend our research to the relationship between adolescents’ perceived quality of financial resources and SHC.

Data regarding the prevalence of SHC in the Greek adolescent population have been drawn mainly from the Health Behaviour in School-aged Children study in 2001/2002 and 2005/2006 [34,35], and have revealed a high prevalence of multiple SHC among Greek adolescents [5,29,34,35]. However, detailed national-specific evidence is missing, since there has been little effort, at least to the best of our knowledge, to explore possible associations with a variety of sociodemographic factors, especially with those that have shown mixed results in international research so far. The main purpose of the present study was to draw a national sociodemographic profile of adolescents’ SHC, by investigating a number of sociodemographic factors. We expected that females and adolescents over 15 years old would report more SHC than their male and younger counterparts. Regarding the impact of adolescents’ area of residence and immigration background on self-reported SHC, our aim was to explore the inconsistent findings that previous relevant studies have supported. Adolescents’ residential area in the form of urban, semi-urban and rural region was assessed for a potential association with SHC. In the same line, we investigated if the trait of immigration in adolescents’ personal or parental background is associated with self-reported SHC. In addition, we anticipated that low socioeconomic status, represented both by parent-reported measures (parental employment and educational status) and by adolescent-specific measures (FAS and PQFR) would be associated with SHC in adolescence. Our final hypothesis was that, among SES assessment measures, the adolescent-specific ones would play the most significant role in predicting adolescents’ SHC.

## Methods

### Participants and procedures

The study was conducted in the year 2003 within the framework of the European project ‘Screening and Promotion for Health Related Quality of Life (HRQoL) in Children and Adolescents: A European Public Health Perspective’ [36]. The school sampling in Greece was random, multistage, and based on the age and sex distribution of school children living in the 54 geographical sectors of the country. The target population was adolescents aged 12 to 18. A sample size of 1,800 adolescents was considered necessary to detect a minimally important difference of half a standard deviation (SD) in Health Related Quality of Life (HRQoL) scores within each age strata between children with and without special healthcare needs or a chronic condition. A response rate of approximately 70% was expected, so the initial sample size was set at 2,400 children and adolescents. In Greece, ages 12 to 18 correspond to six secondary school grades. Approximately 400 students were included from each of the 6 age groups/grades in order to reach the original target of 2,400 adolescents. For example, the total number of students in Greece attending the first grade of the secondary school is 119,055. If an administrative region had a total number of 2,174 students attending the first grade of the secondary school, then 8 students were randomly recruited from a school in that region ((2,174 × 400)/119,055 = 7.60 students). Each age group/grade had been calculated accordingly, for each sector. Schools in each sector were randomly selected by a computer program and students at each selected school were selected randomly from classroom name lists. A sample of 1,900 adolescents (12 to 18 year olds) was recruited. Inclusion criteria for students were: to belong in the age group under study, to be able to read and complete the questionnaire themselves and to consent to take part in the study. Students were asked to complete the questionnaire at school. Ethical approval was attained from the National Ministry of Education. With regard to the proxy survey, parents were asked to fill in the questionnaire at home within a week and return it to school. Inclusion criteria for parents were adequate reading skills and living with the adolescent. Only one parent (voluntarily selected) could participate for each adolescent included in the study. Previous research on the representativeness of the present sample has reported that non-responder interviews showed no significant differences between responders and non-responders with regard to adolescents’ and parents’ general perceived health, parental marital status and highest educational level, and type of residence, indicating that a selection bias is less likely [37]. A total of 1,194 (that is, 63% response rate) of self-reported questionnaires (40.1% boys) were finally returned and 922 of them (77.2%) without missing values (full data) were analyzed.

### Measures

Students were asked to report their sex, date of birth and school grade. Sex was measured in terms of biological sex as reported by the adolescent, female and male. Age was calculated by subtracting the date of birth from the interview date and was classified according to date of birth in two categories: 12 to 15 years and 15 to 18 years. Area of residence was categorized in three types: urban, semi-urban and rural. Immigration background was based on the country of birth. Students were asked to report the country of birth for themselves and for both of their parents. For the purposes of the present study, being born in Greece was coded as ‘native-born’, while being born out of Greece or having a parent born abroad would be indicative of an immigration background.

SES was measured by the use of FAS, as an indicator of family wealth [26]. Based on common indices of material deprivation, the FAS measure comprises four items which are well understood by young people: family car ownership, having their own unshared room, the number of computers at home and times adolescents spent on holidays in the past 12 months. A composite score is calculated for each adolescent based on his/her response to these four items. The FAS was collected from adolescents in seven categories (from 0 the lowest, to 7 the highest FAS category). In our analysis, FAS was recorded into two groups, low FAS level (0 to 3) and medium/high FAS level (4 to 7), since emphasis was put on assessing associations and discrepancies of the low FAS group of adolescents in comparison to wealthier ones. FAS exhibits acceptable psychometric properties and has been widely used in relevant studies [5,38].

Parental education level was assessed through parental reports using the International Standard Classification of Education (ISCED) [39]. The original seven educational levels were codified in the analysis into three categories: low level, primary school (categories 0, 1 and 2); medium level, secondary school (categories 3 and 4); and high level, university degree (categories 5 and 6). Parental employment status originated from parental reporting on his/her occupational status and was codified in the analysis into unemployed or not, for the purposes of the present study. In the analysis, the highest parental level of education and parental unemployment status were used separately to test differences in adolescents’ SHC.

Adolescents’ subjective perception of the quality of available financial resources (PQFR) is a part of the KIDSCREEN-52 generic instrument [36]. This dimension, abbreviated as ‘Finance’, is incorporated into the Social Functioning domain of the KIDSCREEN-52 instrument, which covers the aspects of autonomy and the opportunity to finance and participate in activities [33]. Each adolescent’s PQFR was assessed by three items addressing the recall period of last week: (1) ‘Have you had enough money to do the same things as your friends?’, (2) ‘Have you had enough money for your expenses?’ and (3) ‘Do you have enough money to do things with your friends?’. Each item was rated on a five-point scale (ranging from ‘not at all’ to ‘extremely’ or from ‘never’ to ‘always’) and the subscores were then added together. The sum score was transformed into values between 0 and 100, with higher scores indicating higher perceived quality of financial resources. The Greek version of the Financial Resources dimension has been found to have good reliability properties with Cronbach's α (0.89) [36].

SHC were measured through the Health Behaviour in School-aged Children Symptom Checklist (HBSC-SCL) [40], a self-administered brief screening instrument which indicates the frequency of occurrence of eight common health complaints [41]. Students were asked: ‘In the last 6 months how often have you had the following?’ and the items included were: headache, stomachache, backache, depressed mood, irritability, nervousness, sleeping difficulties, dizziness. Each health complaint was rated on a five-point frequency scale: ‘about every day’ (1); ‘more than once a week’ (2); ‘about every week’ (3); ‘about every month’ (4); ‘rarely or never’ (5). Items were added together to generate an index score of subjective health complaints with minimum value = 8 and maximum value = 40. They were recorded so as higher scores indicated more subjective health complaints. In quantitative analysis the HBSC-SCL has revealed a satisfactory reliability [41] with test-retest reliabilities ranging from 0.70 to 0.80 [12,41]. The Cronbach’s α coefficient in the present study was 0.74.

### Data analysis

Analyses were conducted concerning full data without missing values. Continuous variables are presented with mean and standard deviation while qualitative variables are presented with absolute and relative frequencies. Student t tests and analysis of variance (ANOVA) were computed for the comparison of mean values. Bonferroni correction was used in order to control for type I errors. Pearson correlation coefficient was aimed to explore the association of SHC with PQFR. Data were modeled using multiple linear regression analysis in which the variable indicating SHC was the dependent one. The regression equation included terms for sex, age, area of residence, family socioeconomic background, highest parental education, parental (un)employment status, PQFR and country of birth for adolescents and parents. All categorical variables (sex, age, area of residence, country of birth of adolescents and parents, family socioeconomic background, parental employment and educational status) were treated as dummies in the regression analyses with the reference category cited as in the respective table. PQFR was treated as a continuous variable. Adjusted regression coefficients (β) with standard errors (SE) were computed from the results of the linear regression analyses. Diagnostics for regression models were performed to check if the conditions for regression had been met with the residuals of each model being normally distributed and their variance being constant. Family socioeconomic background and PQFR were examined separately in the linear regression model because model diagnostics with both variables in the model indicated that the regression estimates were highly collinear. Hypothesized interactions of variables in the models were not significant. All *P* values reported are two tailed. Statistical significance was set at 0.05 and analyses were conducted using SPSS statistical software (version 15.0; SPSS, Chicago, IL, USA).

## Results

Sample characteristics are presented in Table 1. Female and younger adolescents (ages 12 to 15 years) were over-represented in the final sample (60.3% and 66.8% respectively). More than 90% of our sample documented Greece as the country of birth for both themselves and their parents (93% of adolescents and 92.1% of their parents). Adolescents were distributed across the three subcategories of area of residence (urban/semi-urban/rural), with a small predominance of the semi-urban area (39.7%). A large number of parents (75.4%) had a medium to high educational level and 95.2% of them were employed. A 37.3% of the participants came from families with a low level of affluence. The mean score of PQFR for our adolescent sample was 70.3 (SD = 24.1), in a scale ranging from 0 to 100.

**Table 1:** Sample characteristics

**Category**	**Value**	**N**	**%**
Sex	Female	556	60.3
	Male	366	39.7
Age in years	12 to 15	616	66.8
	16 to 18	306	33.2
Where does the child live?	Urban	273	29.6
	Semi-urban	366	39.7
	Rural	283	30.7
Family affluence scale	Low	344	37.3
	Medium/high	578	62.7
Highest ISCED category	Low	227	24.6
	Medium	339	36.8
	High	356	38.6
Mother and/or father unemployed?	No	878	95.2
	Yes	44	4.8
Mother and/or father born in other country?	No	849	92.1
	Yes	73	7.9
In which country were you born?	In country	857	93.0
	Other country	65	7.0
Perceived quality of financial resources, mean (SD)	-	70.3 (24.1)	

*ISCED* International Standard Classification of Education.

Table 2 shows the mean SHC score according to all independent factors. Sex, age, FAS, ISCED category and unemployed parent(s) were the factors significantly associated with SHC in the univariate analysis. Female and older adolescents (*P* <0.001), adolescents with low FAS ( *P* = 0.006) as well as those with low educated ( *P* = 0.05) or unemployed parent(s) ( *P* = 0.02) reported a higher mean score of SHC in comparison with males, younger peers, adolescents with medium/high FAS and those with medium/highly educated or employed parent(s). Immigration background was assessed according to country of birth of the adolescent per se or of their parent(s). The analysis showed that adolescents with (a) foreign-born parent(s) reported slightly fewer SHC than the ones with native-born parent(s). In contrast, foreign-born adolescents showed a slight increase of SHC mean score compared to native-born ones. However, the t test associated with both results suggested that there were no statistical significant associations of SHC score with immigration background, neither of the individual per se ( *P* = 0.97), nor of their parents ( *P* = 0.91). What is more, given the small sample size of adolescents with a personal or parental immigration background, it would be safe to support that a genuine association between immigration background and adolescents’ SHC score was not reflected in the present study.

**Table 2:** Mean and standard deviation of the subjective health complaints score and group comparisons according to sample characteristics

**Category**	**Value**	**Subjective health complaints score**		
		**Mean**	**SD**	***P*value**
Sex	Female	18.38	6.24	<0.001^a^
	Male	15.86	5.84	
Age in years	12 to 15	16.63	6.1	<0.001^a^
	16 to 18	18.88	6.17	
Where does the child live?	Urban	17.24	6.48	0.52^b^
	Semi-urban	17.63	6.13	
	Rural	17.18	6.05	
Family affluence scale	Low	18.11	6.41	0.006^a^
	Medium/high	16.95	6.04	
Highest ISCED category	Low	18.04	6.23	0.05^b^
	Medium	17.44	6.38	
	High	16.90	6.00	
Mother and/or father unemployed?	No	15.20	5.44	0.02^a^
	Yes	17.49	6.23	
Mother and/or father born in other country?	No	17.40	6.17	0.91^a^
	Yes	17.17	6.7	
In which country were you born?	In country	17.37	6.15	0.97^a^
	Other country	17.54	7.04	
Perceived quality of financial resources		−0.23^c^		<0.001

^a^Student t test.

^b^Analysis of variance (ANOVA).

^c^Pearson’s correlation coefficient.

*ISCED* International Standard Classification of Education.

After Bonferroni correction it was found that those whose parents belonged to a low ISCED category had a greater SHC score compared to those whose parents belonged to a high ISCED category. What is more, a significant negative association between SHC score and the PQFR scale was found (r = −0.23, *P* <0.001), indicating that higher scores on the PQFR scale are related to fewer SHC.

In tandem, multiple linear regression analyses were conducted (Table 3). Results illustrated that self-reported SHC scores were lower for male participants in comparison to females (*P* <0.001), as well as for younger compared to older adolescents ( *P* <0.001). Also, adolescents with medium/high FAS had fewer SHC compared to those belonging to the low FAS category ( *P* = 0.029). Parental unemployment and ISCED category were not significant predictors in the multivariate model. Additionally, multiple analyses revealed that the PQFR scale was a significant predictor for the SHC scale, indicating that adolescents who reported being more satisfied with their financial resources also reported lower SHC scores ( *P* <0.001).

**Table 3:** Results from multiple linear regression analyses with dependent variable the subjective health complaints score

**Category**	**β**	**SE**	**β**^**a**^	***P*value**
Sex				
Female (reference)				
Male	−2.31	0.41	−0.18	**<0.001**
Age				
12 to 15 (reference)				
16 to 18	1.89	0.44	0.14	**<0.001**
Where does the child live?				
Urban (reference)				
Semi-urban	0.18	0.50	0.01	0.72
Rural	−0.61	0.54	−0.05	0.27
Family affluence scale^b^				
Low (reference)				
Medium/high	−0.94	0.45	−0.07	**0.029**
Highest ISCED category				
Low (reference)				
Medium	−0.33	0.53	−0.03	0.53
High	−0.55	0.57	−0.04	0.33
Mother and/or father unemployed?				
No (reference)				
Yes	1.75	0.94	0.06	0.06
Mother and/or father born in other country?				
No (reference)				
Yes	−0.47	0.91	−0.02	0.61
In which country were you born?				
In country (reference)				
Other country	0.26	0.97	0.01	0.79
Perceived quality of financial resources^b^	−0.06	0.01	−0.22	**<0.001**

^a^Standardized regression coefficients.

^b^Examined separately in the linear regression model adjusted for all other variables.

*ISCED* International Standard Classification of Education.

## Discussion

The main purpose of the present study was to investigate a number of sociodemographic factors and determine the ones that are associated with SHC in a large, random, nationwide sample of Greek adolescents, in an effort to provide national-specific evidence. The sex and age of adolescents were included in our analysis as factors already conceded to demonstrate strong associations with adolescents’ SHC in previous research [7,8,11,12]. Socioeconomic background was assessed by incorporating parent-reported (parental education and employment status) and adolescent-specific measures (FAS and PQFR). To extend our knowledge, we also assessed the potential effect of adolescents’ residential area (that is, urban, semi-urban, rural) and adolescents’ personal or parental immigration background.

### Age and sex

Our findings suggest that sex and age are the factors demonstrating the most constant relationships with SHC reported by adolescents. Females and adolescents over 15 years old reported more SHC than their male and younger counterparts, in accordance with previous studies in this field [5,35,42]. In parallel, age and sex were found to be significant predictors of adolescents’ SHC. The analyses performed revealed significant differences between the categories of each variable, representing a genuine impact of age and sex on adolescents’ reported SHC. However, the actual dimension of the mean SHC score reported in Table 2 calls for caution regarding males and younger peers as well. The fact that male and older adolescents were under-represented in the study sample (approximately 40% and 33% respectively) should not be overlooked, since it may have affected internal correlations.

Focusing on Greece, similar results have been documented regarding recurrent pain complaints of younger schoolchildren [43], as well as worse reported general and mental health by female and older adolescents [44-46]. Explanations for the higher prevalence of complaints in older adolescents, especially females, include age-related changes in biological and cognitive functioning and psychosocial aspects, such as role/gender expectations and societal/family demands [11,12,22,47]. Pubertal timing and maturational processes usher the adolescent into a transitional period, where self-perception, body image and gender identity are modified and readapted. During this challenging time, self-esteem levels often decrease, contributing to an age-progressive deterioration of health perception [42]. In explaining the female predominance in psychosomatic symptoms in adolescence, the role of self-image, represented by self-esteem and body image, is underlined again as a major contributing factor to sex differences in reported symptoms [48]. In parallel, cultural and macro-level societal characteristics are often endorsed in the justification of gender differences in self-reported health, indicating that it is socially acceptable for females to express worries about their health and well-being [49], especially when their environment is characterized by gender inequities in education, occupation, income, political power and life expectancy [12,48].

### Types of area of residence

In discussing adolescents’ areas of residence, our study did not detect any differences in adolescent SHC levels across types of area of residence. Kristjansdottir [50] also reached the same conclusion as early as 1996 in her nationwide study on experienced stomach pain in adolescents, stating that no residential differences were detected. In regards to mental health outcomes, few studies have demonstrated that living both in urban [14] and rural areas [13] is positively associated with mental health symptoms and behavioral problems [51] in adolescence, while lack of differences in urban and rural adolescents’ mental health has also been reported [52]. Our study suggests that Greek adolescents appear to share common health concerns, regardless of the type of area they live in. In fact, this is not the sole such finding coming from Greece. Evidence from recent Greek studies support that type of residence according to level of urbanization had only a weak impact on reported HRQoL in adult population [53] and no impact on adolescent health care use [54]. Our finding supports the argument that neither urbanity nor rurality *per se* is indicative for an increase in adolescents’ SHC. Nonetheless, it requires caution, given the limited focus on regional differences in adolescents’ reports of SHC, at least to the best of our knowledge. Further research is needed to thoroughly explore the association between types of area of residence and adolescents’ SHC as well as to shed light on the unique stressors of urban (that is, lack of social cohesion, violence) and rural life (that is, geographic isolation, loneliness) that may mediate adolescents’ subjective perception of their health and functioning [55].

### Immigration background

Our study suggests that immigration background exerts no effect on adolescents’ SHC, since the differences observed between native adolescents and adolescents with a personal or parental immigration background were small and non-significant. It appeared that adolescents who have been born abroad report slightly more SHC comparing to native-born ones, while adolescents with (a) foreign-born parent(s) report less SHC in reference to their peers who have both parents born in Greece (Tables 2 and 3). Even though both of the aforementioned differences were of low magnitude and with no statistical significance, they could be viewed as an indication of a potential effect of the immigration generational status on adolescents’ reported SHC, suggesting that first and second generation immigrant adolescents are likely to present differently in terms of psychosomatic health. Our aim, however, was to explore if the trait of immigration in adolescents’ personal or parental background is associated with self-reported SHC, thus, we limited our study to this purpose.

Our finding seems to be in line with previous Greek studies that have documented an even distribution of prevalence rates for mental health problems among native and immigrant adolescents in Greece [56-58]. Nonetheless, it adds to a long sequence of inconclusive study outcomes regarding the potential impact of immigration background on adolescent health. There has been evidence suggesting both the presence [59,60] and the absence [61,62] of differences in adolescents’ health, mental health and well-being according to immigrant status. Given the lack of clear, general patterns [63], our finding should not be generalized.

On the contrary, caution is required in its interpretation for two more reasons. First, only a small proportion of the participants in the study were born abroad (N = 65) or had a parent born abroad (N = 73), indicating that the trait of ‘immigration background’ could be attributed to a rather small number of adolescents. Therefore, the present finding was derived by a small sample size, jeopardizing any attempt to extend our conclusions to population level. Second, a mixed definition of immigration background was employed, mainly due to the small sample size described above. ‘Immigration background’ was used as an umbrella term, including adolescents who have been born abroad and native-born adolescents whose parent(s) was/were born abroad, as well as adolescents from different ethnic/minority and cultural backgrounds. This mixed definition was meant to put emphasis on the general immigration process and its impact on adolescent health, rather on specific characteristics of immigrant groups, in order to shed more light on the scarce evidence regarding health outcomes among adolescents in Greece.

Exploring the impact of immigration background on adolescents’ health seems to be a complex, multidimensional task that needs to include various factors occurring at the same time. A recent study [64] focusing on potential differences in perceived health complaints and self-reported health between native and immigrant adolescents showed that discrepancies, albeit detected, were attributed to other factors linked to migratory experience (for example, socioeconomic problems, integration/discrimination, and so on), rather than immigration background itself. As immigration rates are rising, there is a growing need for immigrant-directed studies focusing both on immigrant-specific groups and on the financial, social, relational and psychological factors that have been supported to relate to migratory experience that influence adolescents’ health. [15,16,64].

### Socioeconomic status

With regard to the socioeconomic position that is associated with increased SHC in adolescence, our study supports previous findings [5,29,35], indicating that lack of financial resources is associated with increased self-reported complaints about somatic and psychological health in adolescence. In order to measure SES, we incorporated multiple variables that reflect its multidimensional nature. Previous research has stressed the value of applying measures that describe parental status, family wealth and the child’s subjective experience in order to achieve a holistic view of health inequalities in childhood and adolescence [26]. Our study included parent-reported and adolescent-reported measures. Indicators based on parental reports were parental education and employment status. Indicators deriving from adolescents’ reports were a measure of family wealth (Family Affluence Scale) and the adolescent’s perceived quality of his/her financial resources as a ‘subjective’ indicator of how the individual assesses the possibility to finance and participate in activities with peers.

The univariate analyses showed that both parent and adolescent specific measures of SES were associated with self-reported SHC, as it has been previously supported regarding other measures of self-rated health in adolescence as well. Nevertheless, the multiple linear regression analyses presented different results. The parent-reported measures of parental education and employment status that were used in our study had only a very limited role in predicting adolescents’ SHC, adding, thus, to a long sequence of inconsistent results that investigate the relationship between the ‘traditional’ parent-reported SES indicators and adolescents’ health complaints [23,24,65,66].

Caution is required, though, when interpreting our finding regarding parental employment status due to results revealed by regression analyses (Table 3). Even though the standardized regression coefficient for unemployment was small and non-significant (standardized b = −0.06, *P* = 0.06), the unstandardized regression coefficient (b = 1.75) was one of the highest, after sex (b = −2.31) and age (b = 1.89), indicating that having (an) unemployed parent(s) could serve as a predictor of adolescents’ reported SHC. However, the lack of statistical significance ( *P* = 0.06) could be attributed to the high standard error which, most probably, was due to the small sample size of adolescents with (an) unemployed parent(s). In any case, our result regarding parental unemployment needs further investigation in larger adolescent samples with more unemployed parents involved. Recent research suggests that the relationship between parental unemployment and adolescent poor subjective health is not a linear one. A variety of factors, ranging from the duration of unemployment to individual (that is, mother/father unemployed) and societal (unemployment benefit, reasons of unemployment) ones, are documented to be involved in this complex interplay [65,67].

However, the adolescent-specific measures of family affluence and PQFR proved to have robust associations with SHC among all SES indicators used. FAS made a small but significant contribution to exploring the socioeconomic gradient in adolescents’ SHC, indicating that adolescents coming from low affluent families report increased SHC scores. The use of PQFR revealed significant socioeconomic discrepancies, even though their actual dimension in absolute number was rather small. In fact, analysis (Table 3) revealed that for a unit increase in the PQFR scale, ranging from 0 to 100, there would be a 0.06 decrease in the SHC scale, ranging from 8 to 40. Albeit its low magnitude, this result reflected a genuine impact of the PQFR scale on the SHC score (*P* <0.001), suggesting that the PQFR measure could be a useful indicative tool in capturing socioeconomic differences in the field of adolescent psychosomatic health. What is more, PQFR was the most ‘subjective’ SES measure involved in our study, since it focused exclusively on adolescents’ personal perception. Most importantly, though, this latter measure added an element of self-perceived social comparability by incorporating peers as a comparable social group. Peer influence has been highlighted for its profoundness in adolescence, since norms and values, behaviors and identity transitions are forming and peer pressure may play a crucial role [68]. Considering this, involvement of the peer group in adolescents’ subjective financial perception may be of added value, as a possible source of social pressure. In this way, the adolescents’ view of personal relative financial standing in the peer context was also reflected, accomplishing, thus, their subjective financial perception.

Our finding that PQFR could serve as a significant predictor of subjective health outcomes in adolescence, is in line with other similar findings deriving from adult [31] and adolescent population [30,44,69]. Various similar measures regarding adolescents’ subjective perception of their financial/social status have been tested by researchers in different countries and have demonstrated strong associations with health outcomes [26,30,44,69-71]. Given that adolescence is a period when self-conceptualization matures, perceptions of socioeconomic status may be based on both parental SES and the adolescent’s sense of his or her own standing [32]. Therefore, the need to incorporate adolescent-reported measures when examining the role of socioeconomic conditions in adolescents’ health is underlined. However, since it has been only recently that subjective measures of socioeconomic status are employed in adolescent health research, their further use is required in order to determine their association with specific health outcomes.

### Strengths and limitations

The main strength of the present study was the large, nationwide random sample of adolescent general population. The use of standardized tools lends to our study extra merit. Additionally, it was one of very few studies to explore a variety of sociodemographic determinants of Greek adolescents’ SHC, involving at the same time the use of parent-reported and adolescent-reported indicators of socioeconomic status. However, there are certain limitations that need to be acknowledged. Due to the cross-sectional data, no causal relationships between variables could be drawn. Emphasis should also be placed on some sample considerations. There was a tendency in our sample for a higher response rate from girls compared with boys and from younger participants in relation to older ones. This tendency, however, is commonly met in school-based surveys, and is frequently observed across sampling methods and countries [72]. It should be stressed, however, that the methodology of the European project, within which the present study was conducted, achieved a sufficient degree of representativeness to provide reference population values, as provided elsewhere [72,73]. In discussing sampling issues, the small number of adolescents with immigration background and of adolescents with (an) unemployed parent(s) limits our potential to draw safe conclusions and should, therefore, be considered preliminary. It should be emphasized, however, that our finding concerning immigration background has some additional limitations: (i) it is based on a mixed definition of ‘immigration background’ that does not allow a thorough investigation of specific characteristics of immigrant groups, such as generational status (first, second and third-immigration adolescents), culture of origin, and so on, that have been supported to influence immigration experience [16,74], (ii) there may be immigrant groups in Greece that are not represented in the analyzed school-based sample, and (iii) our study did not extend to control factors (for example, socioeconomic problems, integration/discrimination, and so on) that may be confounded with immigration background. In the same vein, regarding parental employment status, we did not involve factors such as the sex of unemployed parent, even though there has been evidence suggesting that adolescents’ subjective health complaints differ according to paternal or maternal unemployment [23,67,71].

## Conclusions

Exploring the sociodemographic determinants of Greek adolescents’ SHC was the major aim of our study. Socioeconomic inequalities were apparent when using adolescent-reported indicators, such as family wealth and perceived quality of financial resources, but not the traditionally used parent-reported indicators of parental employment status and education level. Therefore, great caution should be placed when exploring socioeconomic inequalities in adolescent health, since different results could derive from parent-based and adolescent-specific measures. Our study highlights the need to incorporate adolescent-reported measures when examining the role of socioeconomic conditions in adolescents’ health. Professionals in health promotion and clinical settings should include such adolescents’ subjective measures in their effort to explore possible sources of stress or social discomfort that may influence adolescents’ complaints about their psychosomatic health, so as adolescents’ experience is more thoroughly assessed. Deeper understanding of how adolescents perceive their lives and living situations in reference to their peers and the impact on their subjective health could lead to health promoting interventions and policies that would target to vulnerable adolescent subpopulations effectively. In discussing adolescence, future research should be directed to shed more light on the developmental changes of the adolescent period that may influence individuals’ perceived social/financial stratification. Further attention should also be placed on defining the factors linked to specific demographic characteristics of social and family life (that is, area of residence, parental unemployment and immigration background) that act as stressors (for example, social capital, neighborhood deprivation, and so on) and may correlate to adolescents’ experience of health complaints.

## Abbreviations

SHC, subjective health complaints; SES, socio-economic status; FAS, Family Affluence Scale; HBSC-SCL, Health Behaviour in School-aged Children-Symptom Checklist; PQFR, Perceived Quality of Financial Resources.

## Competing interests

The authors declare that they have no competing interests.

## Authors’ contributions

DP, GG, CT, CD and GK participated in the preparation of the paper. UR-S coordinated the European project ‘Screening and Promotion for HRQoL in Children and Adolescents - A European Public Health Perspective’. YT had overall supervision of the study. All authors read and approved the final manuscript.
